# Culturable Aerobic and Facultative Anaerobic Intestinal Bacterial Flora of Black Cobra (*Naja naja karachiensis*) in Southern Pakistan

**DOI:** 10.1155/2014/878479

**Published:** 2014-04-09

**Authors:** Junaid Iqbal, Mehwish Sagheer, Nazneen Tabassum, Ruqaiyyah Siddiqui, Naveed Ahmed Khan

**Affiliations:** ^1^Department of Biological and Biomedical Sciences, Aga Khan University, Stadium Road, Karachi 74800, Pakistan; ^2^Department of Pediatrics and Child Health, Aga Khan University, Karachi 74800, Pakistan

## Abstract

Using morphological analysis and biochemical testing, here for the first time, we determined the culturable gut bacterial flora (aerobes and facultative anaerobes) in the venomous Black Cobra (*Naja naja karachiensis*) from South Asia. The findings revealed that these snakes inhabit potentially pathogenic bacteria including *Serratia marcescens*, *Pseudomonas aeruginosa*, *Shewanella putrefaciens*, *Aeromonas hydrophila*, *Salmonella* sp., *Moraxella* sp., *Bacillus* sp., *Ochrobactrum anthropi*, and *Providencia rettgeri*. These findings are of concern, as injury from snake bite can result in wound infections and tissue necrosis leading to sepsis/necrotizing fasciitis and/or expose consumers of snake meat/medicine in the community to infections.

## 1. Introduction

Black Pakistani Cobra (*Naja naja karachiensis*.) is a highly venomous snake that is commonly found in southern parts of South Asia [1]. Like other* Naja* members, this Cobra species found in southern Pakistan produces potent venom, which is a major contributor in snake bite-related deaths in South Asia [2]. Additional complications can arise as snake bite injury can result in wound infections and tissue necrosis leading to sepsis/necrotizing fasciitis [1, 2]. Thus, there is a need for clinicians to consider appropriate antibiotics in the presence of or likelihood of wound infection; however, there is no single report of the bacterial flora of Cobra found in South Asia. In addition to the bacterial flora, several other factors determine the outcome of snake bite wound infection including microbial contamination from the environment, handling of the snake bite, and the health of the patient. Previous studies have determined the oral bacterial flora in Cobra [3]. A wide range of bacteria were isolated including pathogenic coagulase-negative* Staphylococcus, Enterococcus* species,* Morganella morganii*,* Enterobacter* specie*s*,* Pseudomonas*,* Aeromonas hydrophila*,* Citrobacter freundii*,* Proteus* species,* Providencia rettgeri,* and* Clostridium* species. The choice of determining the oral bacterial flora is important and sensible. Goldstein et al. [4] have suggested that the snake oral flora is most likely faecal in nature, as the live prey may defecate in the snake's mouth while being ingested. Later, Lam et al. [3] addressed this concern by determining oral bacterial flora from snakes that were freshly captured from the wild and fasted before sample collection, but even then contamination from prey-bacteria cannot be ruled out. For example, Theakston et al. [5] studied oral swabs from newly captured Malayan pit vipers (*Calloselasma rhodostoma*) and found gut-related Gram negative rods such as* Enterobacter* and* Pseudomonas* species and some staphylococci and Clostridia. Thus, gut bacterial flora may accurately represent the Cobra-associated bacterial flora. Thus, the overall aim of the present study was to determine the gut bacterial flora of Black Pakistani Cobra (*Naja naja karachiensis*) found in southern Pakistan [1].

## 2. Material and Methods

### 2.1. Specimen Collection

During the period of June to October 2012, three Black Cobra snakes were captured from the Interior Sindh (Tharparkar region), Pakistan, and brought to Dow University of Health Sciences for species identification by a qualified team with over 10 years of herpetological experience in the territory. The present study included newly captured live snakes. They were not given any food, drugs, or antibiotics for three days. The snake experts and a veterinary surgeon were responsible for all snake handling. The genus of snake, together with the health condition of the snake was identified by the veterinarian who has extensive expertise of herpetology. As in routine procedures, snakes were anesthetized using chloroform and the surface disinfected with 70% ethanol and subsequently dissected to expose the distal part of the gut. The distal part of the gut from the end of the small intestine and up to cloaca was further dissected open and the greenish semisolid gut contents were collected into 15 mL sterile falcon tubes.

### 2.2. Aerobic Culture

In the laboratory, snake gut contents (~3 to 5 g) were resuspended in 5 mL of phosphate buffered saline (PBS) and approximately one mL was streaked onto nutrient agar plates (100 *μ*L per plate). Two sets of plates were prepared. One set of plates was incubated at 25°C whereas other at 37°C for 48 h. Both sets were incubated only aerobically. Bacterial colonies exhibiting growth upon repeated subculturing on nutrient agar plates were selected from all three snakes and streaked onto nutrient agar plates in order to obtain pure cultures. These pure cultures were maintained in nutrient broth containing 25% glycerol and stored at −80°C until used. These strains were subcultured from freezer on nutrient agar plates and identified.

### 2.3. Bacterial Identification

Bacterial colony characteristics such as shape, margin, elevation, color, opacity, and consistency were recorded. All bacterial cultures were subjected to Gram staining to further characterize them. Gram positive bacterial cultures were tested for spore formation, catalase production, and oxygen tolerance, whereas Gram negative cultures were inoculated on citrate utilization medium, sulfide-indole-motility (SIM) medium, triple sugar iron (TSI) slants, and N,N-dimethyl-*p*-phenylenediamine (DMPD) discs for oxidase test. Based on oxidase activity, bacterial cultures were identified further using analytical profile index (API) strips -E and -NE (BioMérieux Inc.). Oxidase positive cultures were inoculated on API-NE and oxidase negative cultures on API-E strips. The results were then interpreted and bacteria were identified using information provided by the manufacturer (BioMérieux Inc.).* Shewanella putrefaciens* and* Ochrobactrum anthropi *were tested further for DNase production using DNA agar medium (BioMérieux). Identification of* Aeromonas hydrophila* was confirmed using the 0129 disc diffusion test (Oxoid). Production of pyocyanin and pyoverdin pigments from* Pseudomonas aeruginosa* was tested using* Pseudomonas* agar-P and agar-F (BD Biosciences), respectively.* Salmonella* isolates were characterized further using* Salmonella* group specific antisera (Denka Seiken Co.).

## 3. Results

The gut contents of the Black Cobra snake were inoculated onto two sets of nutrient agar plates, which were incubated at 25°C and 37°C, respectively, for 48 h. Nineteen culturable bacterial strains (C1–C19) were selected (based on growth at 25°C as well as at 37°C and repeated culturing), which were conserved among three snakes, while the remaining ones were either unidentifiable, could not be subcultured, duplicate, or doubtful contaminant. Gram staining revealed that all of the bacterial cultures were Gram negative rods, except for C14 which was found to be Gram positive rods with spores. The colony morphology of the bacterial isolates revealed variable morphology as summarized in Table 1. Only few of the bacterial isolates were found to produce pigments. Bacterial isolates encoded C1 and C5 formed bright red and yellow colonies, respectively. Bacterial isolates encoded C2, C8, and C10 produced a brownish pink pigment, which diffused into the medium (Table 1). Based on the morphological and biochemical analysis, nine different bacterial types were identified, that are representative of three snakes (Table 2). Among the nineteen different bacterial isolates tested, five (C1, C3, C12, C16, and C18) were identified as* Serratia marcescens*.* Serratia marcescens *is known to produce a red pigment known as prodigiosin, but only one of the five isolates tested, that is, C1, produced bright red pigment, while the rest of the four isolates were nonpigmented. These results are consistent with previous findings where isolation of nonpigmented* Serratia marcescens *was reported from clinical samples [6]. Three of the bacterial isolates tested (C2, C8, and C10) were identified as* Pseudomonas aeruginosa*. These three strains were positive for the production of the pigments pyocyanin and pyoverdin. Among other isolates, C4, C6, and C7 were identified as* Shewanella putrefaciens*, while C9 and C11 were identified as* Aeromonas hydrophila*, and C13 and C15 were identified as* Salmonella* species. The serological typing results show that both of the* Salmonella* strains belong to the subgroup III (data not shown). The remaining four bacterial isolates tested were identified as follows: C5 was identified as* Moraxella* sp.; C14 was identified as* Bacillus* sp.; C17 was identified as* Ochrobactrum anthropi*; and C19 was identified as* Providencia rettgeri*. Table 2 collates the results of the biochemical analysis and identification of the different bacterial isolates.

## 4. Discussion

The comparative analysis of the snake gut flora that we have isolated with other metazoan gut flora revealed that members of the genera* Bacillus* and* Pseudomonas* are common inhabitant of insects, fishes, amphibians, reptiles, birds, and the mammalian gut [7, 8]. As the Cobra mostly feed on rodents, frogs, toads, small birds, and other small snakes, it is possible that the bacteria from within their prey may colonize the gut of the snake; however, a study in the Burmese Python showed that most of the gut flora of snake was native rather than derived from their prey [8]. Nonetheless, in the present study, snakes were fasted for 3 days to ensure that the gut bacterial flora is not derived from their prey. It is interesting to note that bacterial isolates derived from the Cobra gut were mostly Gram negative (18 out of 19); however, this may not represent the true diversity of the Black Cobra gut microbiota as the culture methods employed in this study tend to facilitate the isolation of aerobes and facultative anaerobes only. For example, previous studies have identified obligate anaerobes and unculturable bacteria from the gut of Burmese Pythons using culture-free molecular techniques [8]. Consequently, future studies can address this issue together with determination of bacterial flora and their antibiotic susceptibility to propose the empirical antibiotic treatment of choice in the initial snake bite management of snakes in South Asia and elsewhere. It is interesting to note that all bacterial isolates identified in this study are known to infect humans. For example, in the case of* Serratia marcescens*, human infections are often associated with the use of contaminated blood products, infusion solutions, and hospital stays [9]. An extensive Canadian community-based study showed that a large fraction of* S. marcescens* infections are community acquired [9]. On the other hand,* Pseudomonas aeruginosa* is an important opportunistic human pathogen that can produce hospital-acquired infections and infections in immunocompromised and cystic fibrosis patients [10]. Similarly* Shewanella putrefaciens*,* Aeromonas hydrophila*,* Ochrobactrum anthropi,* and* Providencia rettgeri* are also opportunistic human pathogens, known to cause different human infections in healthy or immunocompromised or hospitalized patients [11]. As the Black Cobra harbors a number of important human pathogens in their gut, it is tempting to speculate that it may transmit these infectious agents to people who share the same habitat with these reptiles, or during snake bite wound infections, albeit the oral flora of the snake was not determined in the present study. Clinicians should seek the advice of clinical microbiologists to consider appropriate antibiotics in the presence of or likelihood of wound infections. Future studies should determine the oral flora of Black Cobra in order to further investigate the possibility of connection between snake bite and related wound infection. Tribal culture may prove to be additional risk factors and expose local communities to potentially pathogenic bacteria. For example, Cobra meat and organs serve as a source of food and are used in the folk medicine [12]. Based on our findings, it is recommended that users remove the gut part while obtaining snake oil or properly cook the meat before consumption; otherwise, they may risk exposure to different pathogenic bacteria, which may be residing in the gut of snakes.

In summary, for the first time we have shown gut bacterial flora (aerobes and facultative anaerobes) in the venomous Black Cobra from South Asia. The results revealed that these snakes inhabit potentially pathogenic bacteria, which may in turn complicate snake bite wound infections and/or expose consumers of snake meat/medicine in the local community.

## Figures and Tables

**Table 1:** Colonial and cellular characteristics of Black Pakistani Cobra (*Naja naja karachiensis*) bacterial gut isolates.

Isolates	Colonial characters						Cellular characters			
	Shape	Margin	Elevation	Color	Opacity	Consistency	Gram Staining	Shape	Spore Formation	Motility
C1	Round	Smooth	Convex	Red	Opaque	Moist	Negative	Rods	−	+
C2	Round	Irregular	Flat	Brownish pink	Opaque	Moist	Negative	Rods	−	+
C3	Round	Smooth	Convex	Off white	Opaque	Moist	Negative	Rods	−	−
C4	Round	Smooth	Convex	Cream	Opaque	Moist	Negative	Rods	−	+
C5	Round	Smooth	Convex	Yellow	Opaque	Moist	Negative	Rods	−	−
C6	Round	Smooth	Convex	Off white	Opaque	Moist	Negative	Rods	−	+
C7	Round	Smooth	Convex	Off white	Opaque	Moist	Negative	Rods	−	+
C8	Round	Smooth	Flat	Brownish pink	Opaque	Moist	Negative	Rods	−	+
C9	Round	Smooth	Convex	Off white	Opaque	Moist	Negative	Rods	−	+
C10	Round	Smooth	Flat	Brownish pink	Opaque	Moist	Negative	Rods	−	+
C11	Round	Smooth	Convex	Off white	Opaque	Moist	Negative	Rods	−	+
C12	Round	Smooth	Convex	Off white	Opaque	Moist	Negative	Rods	−	−
C13	Round	Smooth	Convex	Off white	Opaque	Moist	Negative	Rods	−	+
C14	Round	Irregular	Flat	White	Opaque	Dry	Positive	Rods	+	ND
C15	Round	Smooth	Convex	Off white	Opaque	Moist	Negative	Rods	−	+
C16	Round	Smooth	Convex	Off white	Opaque	Moist	Negative	Rods	−	−
C17	Round	Smooth	Convex	Off white	Opaque	Moist	Negative	Rods	−	+
C18	Round	Smooth	Convex	Off white	Opaque	Moist	Negative	Rods	−	−
C19	Round	Smooth	Convex	Off white	Opaque	Moist	Negative	Rods	−	+

ND: not determined.

**Table 2:** Biochemical characteristics and identification of Black Pakistani Cobra bacterial gut isolates.

Isolates	GRAM stain	API result	Organism
C1	Gram negative rods	5304721 87.6%	*Serratia marcescens* (red pigmentation)
C2	Gram negative rods	Not set	*Pseudomonas aeruginosa* (green pigmentation)
C3	Gram negative rods	5304721 87.6%	*Serratia marcescens *
C4	Gram negative rods	1011344 99.8%	*Shewanella putrefaciens *
C5	Gram negative rods	0000004 83.2%	*Moraxella* spp.
C6	Gram negative rods	1010154 99.9%	*Shewanella putrefaciens *
C7	Gram negative rods	1011344 99.8%	*Shewanella putrefaciens *
C8	Gram negative rods	Not set	*Pseudomonas aeruginosa* (green pigmentation)
C9	Gram negative rods	7246124 89.3%	*Aeromonas hydrophila *
C10	Gram negative rods	Not set	*Pseudomonas aeruginosa* (green pigmentation)
C11	Gram negative rods	7575755 99.9%	*Aeromonas hydrophila *
C12	Gram negative rods	5306721 96%	*Serratia marcescens *
C13	Gram negative rods	6704512 77.8%	*Salmonella* group C3
C14	Gram positive rods (Spore forming)	Not set	*Bacillus* species
C15	Gram negative rods	6704512 77.8%	*Salmonella* group C3
C16	Gram negative rods	5304721 87.6%	*Serratia marcescens *
C17	Gram negative rods	0041354 98.6%	*Ochrobactrum anthropi *
C18	Gram negative rods	5304721 87.6%	*Serratia marcescens *
C19	Gram negative rods	274310 99.9%	*Providencia rettgeri *
